# Characterization of the Interaction between Cadmium and Chlorpyrifos with Integrative Techniques in Incurring Synergistic Hepatoxicity

**DOI:** 10.1371/journal.pone.0059553

**Published:** 2013-03-14

**Authors:** Liqun Chen, Guangbo Qu, Xue Sun, Shuping Zhang, Lei Wang, Nan Sang, Yuguo Du, Jun Liu, Sijin Liu

**Affiliations:** 1 State Key Laboratory of Environmental Chemistry and Ecotoxicology, Research Center for Eco-Environmental Sciences, Chinese Academy of Sciences, Beijing, China; 2 College of Environment and Resource, Shanxi University, Taiyuan, Shanxi, China; National Cancer Institute at Frederick, United States of America

## Abstract

Mixture toxicity is an important issue for the risk assessment of environmental pollutants, for which an extensive amount of data are necessary in evaluating their potential adverse health effects. However, it is very hard to decipher the interaction between compounds due to limited techniques. Contamination of heavy metals and organophosphoric insecticides under the environmental and biological settings poses substantial health risk to humans. Although previous studies demonstrated the co-occurrence of cadmium (Cd) and chlorpyrifos (CPF) in environmental medium and food chains, their interaction and potentially synergistic toxicity remain elusive thus far. Here we integrated the approaches of thin-layer chromatography and ^1^H NMR to study the interaction between Cd^2+^ and CPF in inducing hepatoxicity. A novel interaction was identified between Cd^2+^ and CPF, which might be the bonding between Cd^2+^ and nitrogen atom in the pyridine ring of CPF, or the chelation formation between one Cd^2+^ and two CPF molecules. The Cd-CPF complex was conferred with distinct biological fate and toxicological performances from its parental components. We further demonstrated that the joint hepatoxicity of Cd ion and CPF was chiefly due to the Cd-CPF complex-facilitated intracellular transport associated with oxidative stress.

## Introduction

The co-existence of various pollutants in environment and food chains is considerably concerned due to the fused impact on environment and public health [1], [2], especially if the joint toxicity of pollutants poses adverse health effects on humans [2]. Mounting evidence suggests that simultaneous exposure of pollutants on organisms can potentiate the toxicity of individual components [3], [4]. Thus far, it has been difficult to study the joint toxicity of pollutants, in particular to determine the interaction between compounds, due to limited technical approaches [5]. Only limited interactions have been fully characterized, such as the interaction of chlorpyrifos (CPF) with methyl mercury [6].

The heavy metal cadmium (Cd) has broad industrial applications, such as battery production and electroplating, and it is substantially dispersed in the environment [7], [8]. Excretion of Cd ion in human body is about 1–2 µg/day, and the half-life is 20–30 years. Exposure to Cd could cause serious diseases, such as itai-itai disease or even cancers [9]. As a typical environmental hazard, Cd is ranked eighth within the top 20 in the priority;list of hazardous substances by the ATSDR (Agency for Toxic Substances and Disease Registry) [10]. CPF is one of the most widely used organophosphoric insecticides worldwide under agricultural and residential settings in the last few decades [11], [12]. Although CPF has been banned for very long time, it still largely remains in water, air and soil, as well as in many dwellings. CPF was found in 100% of indoor air samples and 64–70% of blood samples from mothers and newborns [13]. Large amount of CPF can cause acute toxicity, and even a trace amount of CPF can induce neurological toxicity in fetuses and children [13]. Cd ion and CPF are often jointly present in the same environmental media and food chains, and are simultaneously exposed on organisms [14], [15], leading to pronounced environmental and health problems [16]. They incur common sensitive targets of toxicity, such as carcinogenicity and hepatoxicity [17], [18], [19], [20], and oxidative stress is assumed to be the principal molecular basis underlying cytotoxicity caused by Cd and CPF [21]. Despite the co-occurrence of these two chemicals in environmental medium and food chains, their toxicity and human risk assessment were predominantly based on the toxicological performances of single chemical. The interaction of Cd ion with other organophosphorus pesticide (such as fenitrothion) has been suggested by other studies [22]; whereas the toxic effects of CPF have also been demonstrated to be modulated by metals, such as zinc [23]. However, the synergistic interaction between Cd ion and CPF has not been established thus far, and the corresponding molecular mechanism is largely unknown as well.

In the current study, to elucidate the reciprocal impact between CPF and Cd ions, we here addressed their joint hepatoxicity using a few *in vitro* assessments in a representative human hepatocyte cell line Hep G2. We embarked on their synergistic molecular interaction by integrating techniques, such as thin-layer chromatography (TLC) and ^1^H NMR. Overall, we demonstrated the formation of the Cd-CPF complex, which was conferred with distinct biological fate and toxicological performances from its parental chemicals.

## Results and Discussion

**Figure 1:**
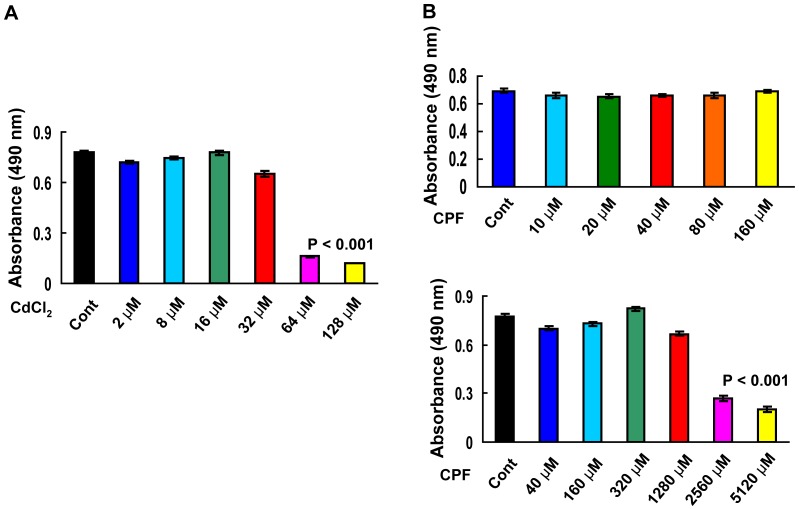
The MTT-based cell survival assay of Hep G2 cells exposed to various concentrations of Cd^2+^ or CPF. The cell viability was assessed by the MTT assay in Hep G2 cells treated with up to 128 µM Cd^2+^ (a) and up to 5,120 µM CPF (b) after 24 h exposure (n = 6).

**Figure 2:**
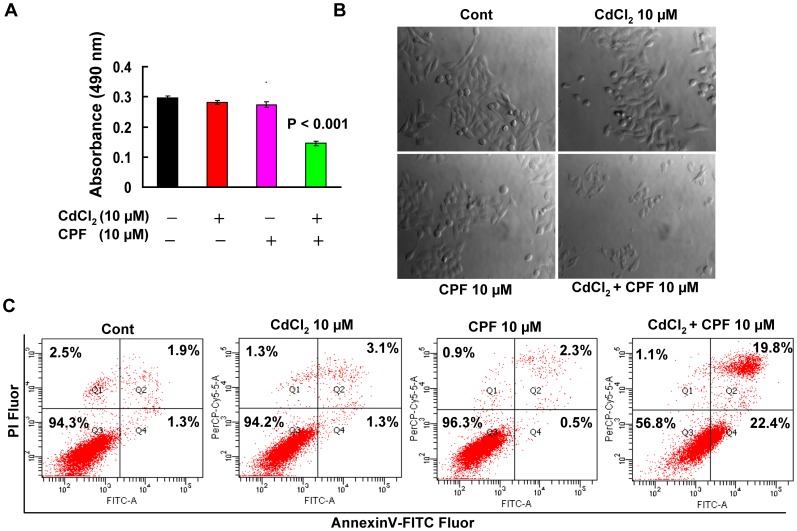
The concomitant exposure of Cd^2+^ and CPF resulted in toxicity to Hep G2 cells. (a) The MTT assay of cells treated individually or jointly with Cd^2+^ and CPF at 10 µM for 24 h (n = 6). (b) The phase-contrast images showed the alternations to the cellular morphology upon Cd^2+^ and CPF treatment. The original magnification was 200×. (c) The representative images of FACS analysis of cell death upon single or joint exposure to Cd^2+^ and CPF for 24 h using the FITC-conjugated Annexin V and PI staining. There were three biological replicates for each group (n = 3).

To evaluate the potential synergistic effect, we first evaluated the joint cytotoxic effect of Cd^2+^ and CPF on Hep G2 cells by assessing the cell viability with the MTT assay. After 24 h, no toxicity was observed to Hep G2 cells treated with up to 32 µM Cd^2+^ and up to 1,280 µM CPF, respectively (Fig. 1a&b). To intensively study the synergistic effect, we chose the concentration of 10 µM for both Cd^2+^ and CPF, at which neither of them caused damage to cell viability (Fig. 1 and Fig. 2a). It should be noted that CPF was dissolved in DMSO and the concentration of 10 µM CPF in culture medium contained only 0.001% DMSO which caused no toxicity to cells compared to the blank control (data not shown). The concomitant exposure of the Cd^2+^ and CPF mix at 10 µM exerted great impairment to Hep G2 cells, causing approximately 50% reduction in cell viability, compared to the control or the individual treatment by Cd^2+^ or CPF (Fig. 2a, P<0.001). Moreover, remarkable morphological alternations representing cell death were observed for the cells upon the combined treatment, as these cells became rounder and smaller than the cells without treatment or treated with only one chemical (Fig. 2b). The FACS analysis with FITC-conjugated Annexin V and PI staining further validated cell death in Hep G2 cells, as largely increased apoptosis (>10 fold increase for the Annexin V^+^ cell population) was detected in cells treated with combined Cd^2+^ and CPF treatment compared to individual Cd^2+^ or CPF treatment (Fig. 2c, P<0.001). These results together demonstrated a strong synergistic cytotoxic effect of Cd^2+^ and CPF on Hep G2 cells.

**Figure 3:**
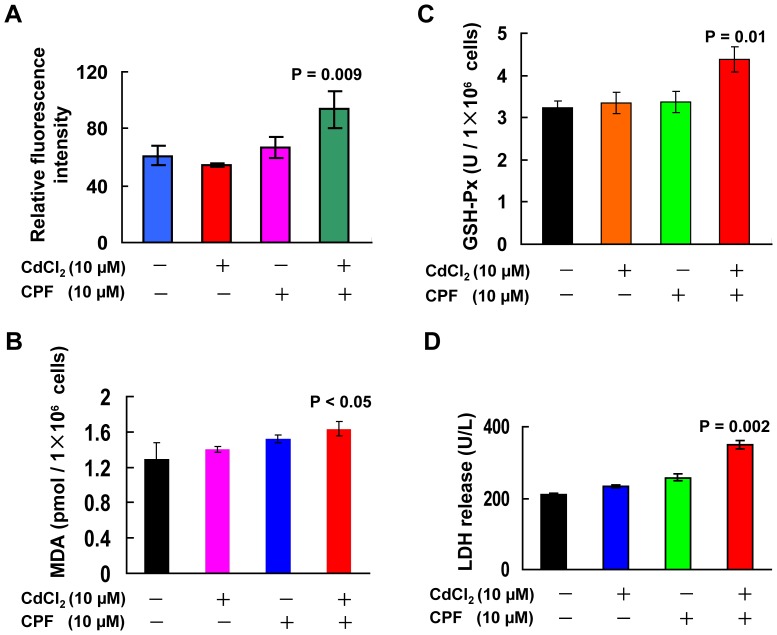
The synergistic hepatoxicity of Cd^2+^ and CPF. Hep G2 cells were treated individually or jointly with Cd^2+^ and CPF both at 10 µM for 6 h (n = 3–4). Thereafter, the intracellular ROS generation (a), MDA level (b), GSH-Px activity (c) and LDH release (d) were assessed, respectively.

Previous studies have documented that both Cd^2+^ and CPF could induce cytotoxicity through oxidative stress, such as reactive oxygen species (ROS) generation [21]. We thus assessed intracellular ROS content in cells upon the concomitant exposure. As shown in Fig. 3a, the ROS level was not significantly changed in Hep G2 cells upon treatment with single compound; however, the ROS level was elevated upon combined exposure compared to the single exposure and the vehicle control (P<0.01). In agreement with previous observations [24], the accumulation of intracellular ROS was likely responsible for the increased cell death caused by the concomitant exposure of Cd^2+^ and CPF. Lipid peroxidation is also an important index in characterizing oxidative stress [25], and malondialdehyde (MDA) is recognized as a marker for lipid peroxidase [26]. For instance, Cd^2+^ *in vivo* administration causes pronounced hepatic oxidative stress in animals, and leads to remarkable liver damage characterized by increased lipid peroxidation and altered antioxidant enzymatic activity [27], [28]. The MDA content was not significantly changed in cells treated with individual component, whereas it was increased upon the joint exposure (Fig. 3b, P<0.05). In response to oxidative stress, the anti-oxidation system is normally enhanced to protect cells against oxidant damage in hepatocytes [27], [29], of which the antioxidant enzyme glutathione peroxidase (GSH-Px) is an important ROS scavenger. The GSH-Px activity was significantly increased only in Hep G2 cells treated with both Cd^2+^ and CPF (P<0.05), compared to the control or the individual treatment by Cd^2+^ or CPF, but not in cells treated with single component in comparison to the control (Fig. 3c). Additionally, we assessed the level of lactate dehydrogenase (LDH), a soluble cytosolic enzyme released into culture medium due to damaged plasma membrane [30]. As shown in Fig. 3d, the LDH level in the culture supernatant was proportional to the results of cell viability as described in Fig. 1 and Fig. 2, as the LDH release was significantly increased in cells upon the binary exposure of Cd^2+^ and CPF only (P<0.01), compared to the control or the individual treatment by Cd^2+^ or CPF. These results together suggested that the concomitant treatment of Cd^2+^ and CPF led to synergistic impairment to Hep G2 hepatocytes linked to oxidative stress, while Cd^2+^ or CPF alone at the same concentration could not bring harm to these cells.

**Figure 4:**
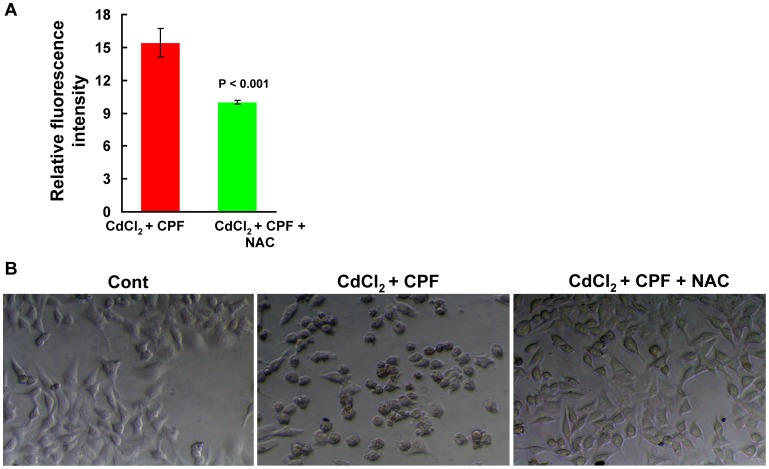
The amelioration of the synergistic hepatoxicity conducted by Cd^2+^ and CPF by the pre-treatment of NAC. Hep G2 cells were treated individually or jointly with Cd^2+^ and CPF both at 10 µM for 6 h, and then the intracellular ROS generation (a) was assessed (n = 4). (b) The representative phase-contrast images. The original magnification was 200×.

To substantiate the role of oxidative stress in the conduction of the synergistic toxicity by Cd^2+^ and CPF, we pre-treated the cells with N-acetyl cysteine (NAC), an antioxidant [31]. As shown in Fig. 4a, induction of ROS was significantly undermined by the pre-treatment of NAC in cells treated with Cd^2+^+CPF, compared to the cells without the pre-treatment of NAC (P<0.001). Therefore, the alterations to the cell morphology were greatly ameliorated upon the pre-treatment of NAC, as the number of the rounder and smaller cells decreased compared to the cells without the pre-treatment of NAC (Fig. 4b). To this end, oxidative stress is demonstrated to be the primary modulator of synergistic impairments mediated by Cd^2+^ and CPF, coupled with reduced cell viability and cell death.

**Figure 5:**
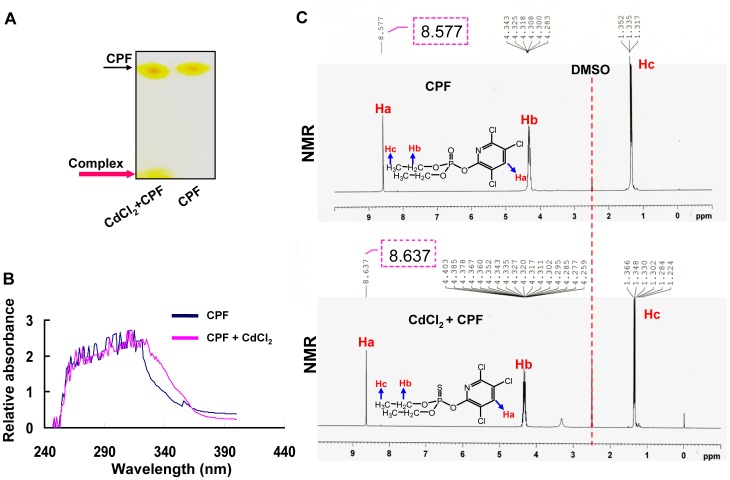
The characterization of Cd^2+^ and CPF interaction. (a) Molecular interaction analysis of Cd^2+^ and CPF was determined by TLC. (b) The UV-vis absorption spectrum analysis of CPF and Cd^2+^ reaction mix. (c) The NMR spectroscopy identified the reaction between Cd^2+^ and CPF. The representative images of the NMR spectra indicated that Cd ion caused great chemical shift of Ha in the pyridine ring of CPF, which decreased the electron cloud density of nitrogen atom and sulphur atom.

**Figure 6:**
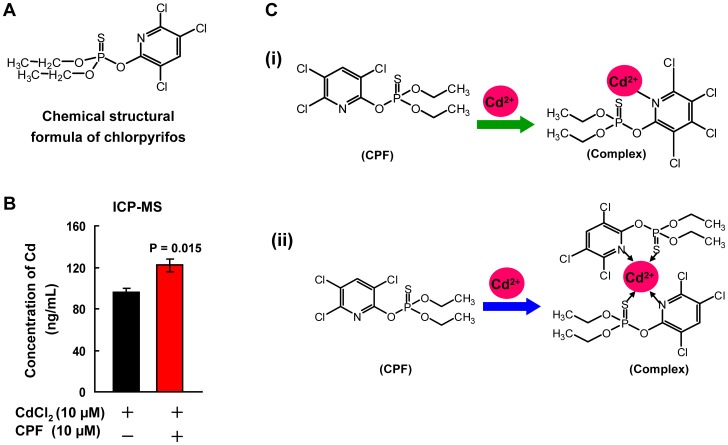
The facilitated intracellular transport of Cd^2+^ through the Cd-CPF complex. (a) The chemical formula of CPF. (b) The intracellular Cd concentrations in cells treated with Cd^2+^ or Cd^2+^+CPF (both at 10 µM) for 24 h assessed by ICP-MS (n = 3). (c) A schematic delineating the molecular interaction between Cd^2+^ and CPF. The interaction between Cd^2+^ and CPF might be the bonding between Cd^2+^ and nitrogen atom in the benzene ring of CPF (i), or the chelation between one Cd^2+^ and two CPF molecules (ii).

To investigate the mechanism of the synergistic toxicity conducted by Cd^2+^ and CPF, we characterized the molecular interaction between these two compounds. The TLC results indicated the existence of CPF and the CPF-Cd complex (Fig. 5a). The CPF-Cd complex presented a separate band as the pink arrow indicated on the TLC plate, while the CPF alone did not. The UV-vis absorption spectrum analysis of the reaction mix also revealed distinct absorption peaks around 320–350 nm between CPF and CPF+Cd, suggesting the formation of CPF-Cd complex (Fig. 5b). Furthermore, the NMR spectroscopy was employed to illustrate the precise site of binding between CPF and Cd. As the the NMR spectra shown in Fig. 5c and Table 1, Cd ions induced great chemical shift of Ha in the pyridine ring of CPF, resulting in decreased electron density of nitrogen atom and sulphur atom (the molecular formula of CPF was presented in Fig. 6a). This observation demonstrated that a new coordination mode was formed between Cd^2+^ and CPF due to the nephelauxetic effect that refers to a decrease in the Racah interelectronic repulsion parameter. To study the proportion of CPF and Cd^2+^ in the complex, we performed the ^1^H NMR spectra using different ratios of CPF to Cd^2+^, *i.e.* 10∶1, 2∶1, 1∶1, 1∶2, 1∶5 at molar concentrations. A significant change in chemical shift was detected at the ratio of 2∶1 for CPF/Cd^2+^ (Fig. 5c and Table 1), suggesting that CPF and Cd^2+^ tended to form a complex with two CPF molecules to one Cd^2+^. We also carried out the NMR spectroscopy at different time points, 6 h, 24 h and 48 h, and no difference in chemical shift of hydrogen atom was observed along time course (Table 1), implying that the complex was quickly formed and stably existed. Similar to this finding, a previous study using the approach of NMR also demonstrated that Hg^2+^ could associated with the two sulfur atoms in the Demeton S side chain, resulting in stabilizing the Demeton S molecule, another organophosphorus pesticide [32]. These data collectively confirmed the formation of a novel complex between Cd ion and CPF.

**Table 1:** Time-dependent ^1^H chemical shift changes of CPF with various ratios of added Cd^2+.^

Time (h)CPF/Cd	0			6			24			48		
	Ha(ppm)	Hb(ppm)	Hc(ppm)	Ha(ppm)	Hb(ppm)	Hc(ppm)	Ha(ppm)	Hb(ppm)	Hc(ppm)	Ha(ppm)	Hb(ppm)	Hc(ppm)
**1/0**	8.577	4.313	1.335	8.577	4.313	1.335	8.577	4.313	1.335	8.577	4.313	1.335
**10/1**	8.635	4.320	1.346	8.634	4.322	1.346	8.634	4.320	1.347	8.634	4.308	1.346
**2/1**	8.637	4.320	1.330	8.634	4.322	1.347	8.637	4.330	1.347	8.637	4.333	1.348
**1/1**	8.621	4.318	1.338	8.636	4.319	1.348	8.619	4.307	1.337	8.620	4.315	1.337
**1/2**	8.627	4.314	1.342	8.628	4.319	1.343	8.627	4.313	1.343	8.627	4.313	1.343
**1/5**	8.551	4.274	1.296	8.549	4.270	1.294	8.548	4.270	1.294	8.549	4.270	1.294

Previous studies demonstrated that the formation of a complex between chemicals could often accelerate their transport across cell membrane and increase the intracellular accumulation [6], [33], likely resulting in cytotoxicity that might not happen to a single chemical [2], [34]. A representative interaction of CPF was identified with methyl mercury, and the formation of this complex significantly enhanced the bioaccumulation of methyl mercury and induced greater toxicity [6]. We thus assessed the intracellular Cd content in Hep G2 cells upon Cd^2+^ or Cd^2+^+CPF exposure both at 10 µM for 24 h. The ICP-MS data indicated that the intracellular Cd concentration in Cd^2+^+CPF-treated cells was increased by >20% (Fig. 6b, P<0.05), suggesting facilitated transport of Cd^2+^ into cells aided by the complex. CPF is a lipophilic molecule that could readily permeate and penetrate the lipid bilayer membrane [35], whereas the transport of Cd ions is rather difficult, and limited transportation mainly relies on two paths, the cell surface sulfhydryl ligand and the calcium channel [36]. Metallothionein (MT) was reported to be induced by Cd and sequesters intracellular free Cd ions via formation of an MT-Cd complex [10]. MT is also an important intracellular component as ROS scavenger, whose suppression could lose the ability to protect cells from ROS-mediated impairment. Meanwhile, CPF exposure was demonstrated to incur a significant reduction of MT content [37]. Since ROS is the predominant cause of apoptosis, MT reduction caused by CPF might enhance ROS-induced cytoxicity [38]. Whether CPF-Cd complex also diminishes MT warrants detailed investigation. To this end, we could speculate that the formation of CPF-Cd complex therefore potentiated their localization and retention inside cells with the aid of CPF-mediated transportation through diffusion crossing the lipophilic membrane, which presumably accounted for the increased oxidative stress and reduced cell viability.

Organisms are typically exposed to a mix of chemicals, where the toxicological action for each individual chemical might be altered by the co-occurring ones. Both Cd ions and CPF are serious environmental pollutants worldwide and they are often found to co-exist in the environment, food, wild organisms and even in human specimens [14], [15]. In the current study, we mechanistically demonstrated a novel interaction between Cd ion and CPF through the binding of Cd^2+^ to the pyridine ring of CPF. One possibility is the bonding between Cd^2+^ and nitrogen atom in the pyridine ring of CPF (Fig. 6c–i); meanwhile the other possibility is the chelation among one Cd^2+^ and nitrogen atom and sulphur atom from two CPF molecules (Fig. 6c–ii). The formation of Cd-CPF complex largely changed their biological fate and toxicological performance with facilitated cellular uptake and increased toxicity to hepatocytes via oxidative stress. These data together verify the molecular mechanism underlying the Cd/CPF-conducted joint hepatoxicity.

This report presents novel results on the toxic effects of a binary mixture of Cd and CPF on HepG2 cells. The binary mixture has barely been studied yet, and not in HepG2 cells in any case. This study is therefore novel in this sense. In deed, these data, for these chemicals that are indeed widely present in the environment, are very useful. The data indicates that CPF may complex with Cd to facilitate its entry into cells, thereby increases the level of Cd in the cells and also its toxic effect through oxidative stress. This mechanism could occur widely for more combinations of other chemicals. Additionally, we addressed the interaction between Cd ions and CPF by integrating the techniques, such as TLC and ^1^H NMR, which would pave the way for future studies in addressing the synergistic interaction between pollutants.

## Materials and Methods

### Chemicals, Reagents and Cell Culture

Chlorpyrifos (CPF) was purchased from Shuangma Fine Chemical Co., Ltd., Nantong City with the purity of more than 99.99%, and Cd^2+^ (in CdCl_2_) was purchased from Sigma. The human hepatoma Hep G2 cells (purchased from the Shanghai Cell Bank of Type Culture Collection of CAS) were cultured in 1640 medium (Hyclone), supplemented with 10% fetal bovine serum (Gibco) and 100 U/mL penicillin/streptomycin (Gibco) in a humidified atmosphere with 5% CO_2_ at 37°C. The stock solutions of Cd^2+^ (in CdCl_2_) and CPF were made in sterile ddH_2_O and DMSO, respectively, and then filtered through Minisart filters (0.45 µm).

### Cell Survival Assay

Cell survival was assessed by the MTT assay following the instructions from the manufacturer (Roche). Briefly, Hep G2 cells were serum starved for 12 h, and were then inoculated into 96-well plates at a concentration of 5.0×10^3^ cells/well upon different treatments. Cells were cultured for another 24 h, and 20 µL MTT (5 mg/mL) was added to each well followed by incubation for 4 h. Thereafter, 200 µL DMSO was added into each well, and the 96-well plates were read at 490 nm on a microplate reader (Thermo) after shaking.

### ROS Detection and LDH Leakage Assay

Hep G2 cells were seeded in 6-well plates overnight and these cells were treated individually or jointly with Cd^2+^ and CPF both at 10 µM for 6 h. The generation of intracellular ROS was spectrophotometrically measured using dichlorofluorescein-diacetate (DCF-DA, Sigma) as described previously [39]. Relative fluorescence intensity was recorded using a fluorescent plate reader (Thermo) at an excitation wavelength of 485 nm and emission was measured at a wavelength of 530 nm. The fluorescence intensity was assayed, which was proportional to the amount of intracellular ROS concentration. For experiments with NAC, cells were pre-treated with 500 µM NAC (Sigma) 1 h prior to the treatment of Cd^2+^+CPF both at 10 µM, and cultured for another 6 h in the presence of NAC. The CytoTox-ONETM Homogeneous Membrane Integrity Assay Kit (Promega) was used to assess LDH release, according to the manufacturers’ instructions. This assay was on the basis of the conversion of lactate to pyruvate in the presence of LDH with parallel reduction of NAD.

### The Cell Death Analysis

Hep G2 cells were seeded in 6-well plates at a density of 3.0×10^4^ cells per well for 24 h. Cells were treated with Cd^2+^ and/or CPF for 6 h, and were then collected after wash with PBS. The proportions of apoptosis and necrosis were determined by the flow cytometry analysis after Annexin V and propidium iodide (PI) staining (BD Biosciences) as previously described [39], [40].

### Assays for MDA Level and GSH-Px Activity

Hep G2 cells were treated individually or jointly with Cd^2+^ and CPF both at 10 µM for 6 h, and thereafter cells were collected into RIPA lysis buffer after wash with PBS. The MDA level and the GSH-Px activity in the Hep G2 cells were assessed according to the manufacturer’s instructions (both from Wuhan Xinqidi Biological Technology Co., LTD, China). Briefly, supernatants of cell lysates were added into pre-coated GSH-Px or MDA monoclonal antibody microelisa wells followed by the conventional procedure as described previously [41].

### Cd Determination through Inductively Coupled Plasma Mass Spectrometry (ICP-MS)

Hep G2 cells in 10 cm-plates (1.0×10^6^) were treated with Cd^2+^ or Cd^2+^ plus CPF both at 10 µM for 24 h. These Hep G2 cells were washed repeatedly with PBS before the collection into digestive solution. The intracellular Cd mass was measured using the ICP-MS method according to the protocol described in a previous study [42]. Briefly, samples were quantified by volume and digested with strong oxidation-acid solution (a mix of nitric acid and hydrogen peroxide with a proportion of 3∶2) overnight. Then, the primarily digested samples were digested thoroughly at 180°C for 20 mins by microwave assisted digestion (MAD, Mars5 HP500, CEM Corporation, USA). Cd concentrations in these samples were finally quantified using ICP-MS (Agilent 7500, USA).

### Thin-layer Chromatography

Chemical-chemical interaction assessment between CPF and Cd^2+^ was performed with the approach of TLC. CPF (0.01 M) and Cd^2+^ (0.01 M) were allowed to react in ethyl acetate or deionized water under slow mixing for 24 h. The mixture was resolved on silica plates using 30% ethyl acetate/70% hexane, and was visualized by exposure to iodine and ultraviolet (UV) light.

### The^ 1^H Nuclear Magnetic Resonance (NMR) Spectroscopy Analysis

The CPF and Cd^2+^ were diluted with d6-DMSO in 5 mm precision NMR tubes. The ^1^H chemical shifts were referenced internally to the solvent resonance. After completely dissolved, samples were placed in the spectrometer for hydrogen spectrum analysis. ^1^H NMR was measured on the 400 MHz spectrometers (NMR in d6-DMSO). Chemical shifts (δ) were given in ppm relative to residual solvent (d6-DMSO; δ 2.50 for 1 H NMR).

### Statistical Analysis

One-way analysis of variance (ANOVA) was used to analyze the mean differences among groups compared to the control. Two-tailed Student's *t* test was used to analyze experimental data between two groups. Data were shown in mean ± SD. P<0.05 was considered statistically significant.
